# Supplementary material for: Alphaherpesvirus gB Homologs Are Targeted to Extracellular Vesicles, but They Differentially Affect MHC Class II Molecules
Source: Viruses. 2020 Apr 10;12(4):429. doi: 10.3390/v12040429 (PMC7232241; doi:10.3390/v12040429)
Supplement: Supplementary file 1 [file viruses-12-00429-s001.zip › viruses-730753 REVISED SUPPLEMENTARY FIGURES.pdf]

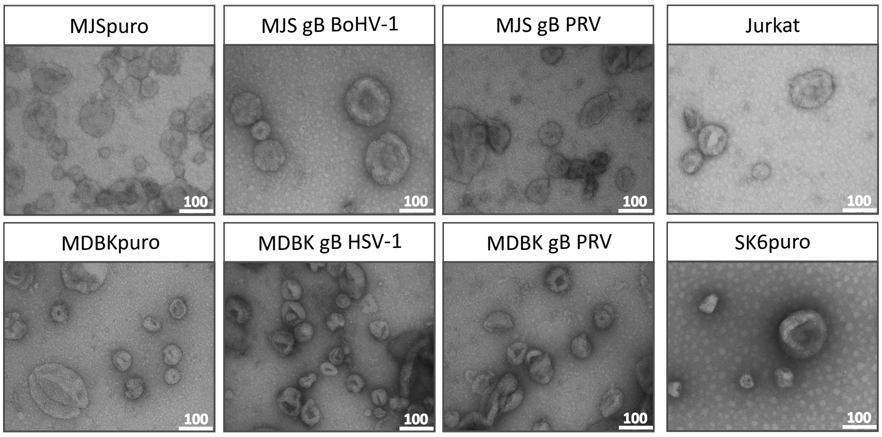

Supplementary Figure S1. Transmission electron microscopy characterization of size exclusion (SEC)-purified extracellular vesicles (EVs) from the constructed gB-expressing stable cell line supernatants. Representative transmission electron microscopy images of EVs preparations are shown; scale bar 100 nm.

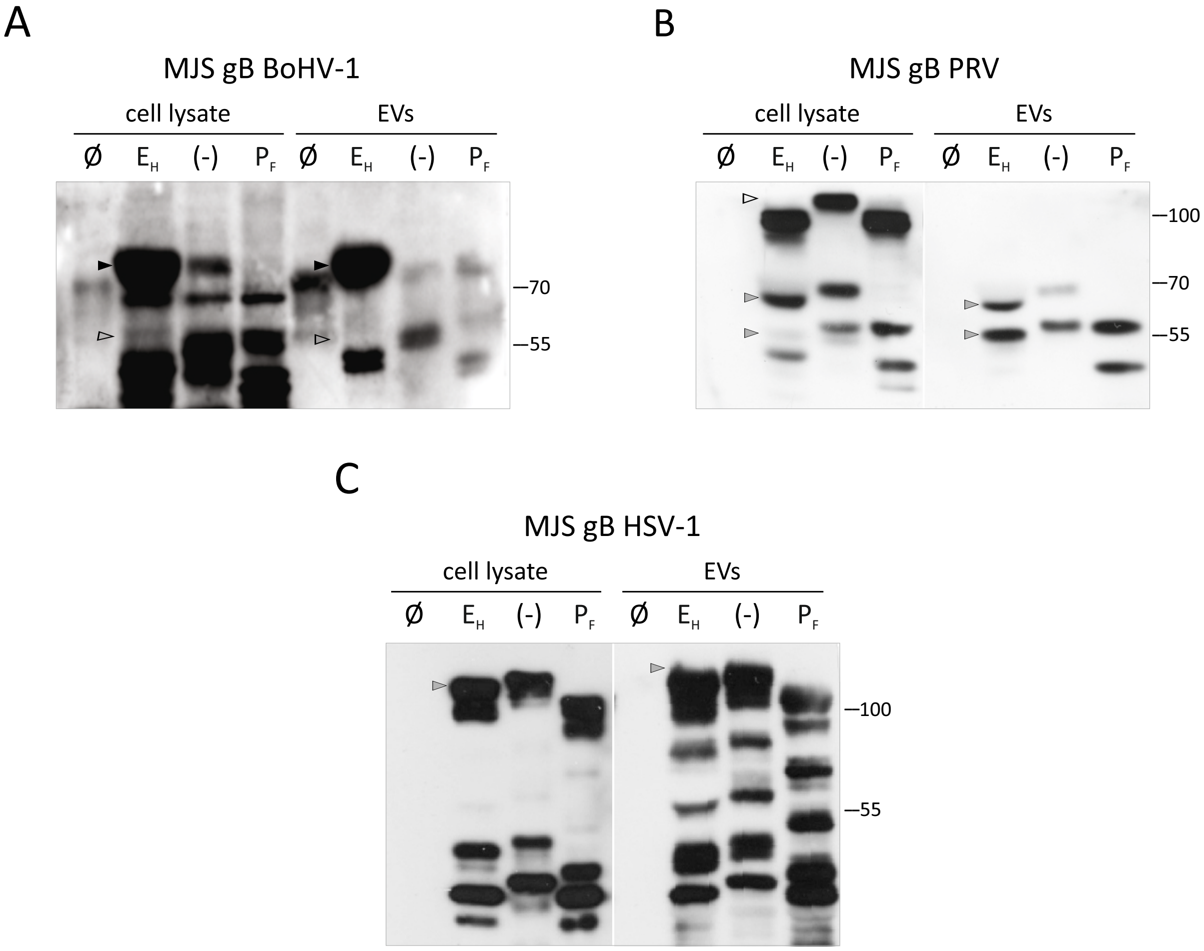

Supplementary Figure S2. Analysis of gB N-glycosylation. MJS gB cell lysates (cl) or EVs lysates were mock-treated (-), treated with endoglycosidase H (EH) or peptide:N-glycosidase F (PF), and subsequently immunoblotted for: (A) BoHV-1 gB with anti-BoHV-1 serum; (B) PRV gB with polyclonal gB-specific antibodies; (C) HSV-1 gB with anti-gB monoclonal antibodies (mAb). MJSpuro, MDBKpuro, and SK6puro (designated as Ø) cell lysates or EVs were used as controls. Full black arrows indicate EH-resistant gB forms; grey-filled arrows represent partially-resistant forms and open arrows indicate EH-sensitive forms. Size markers are in kilodaltons.

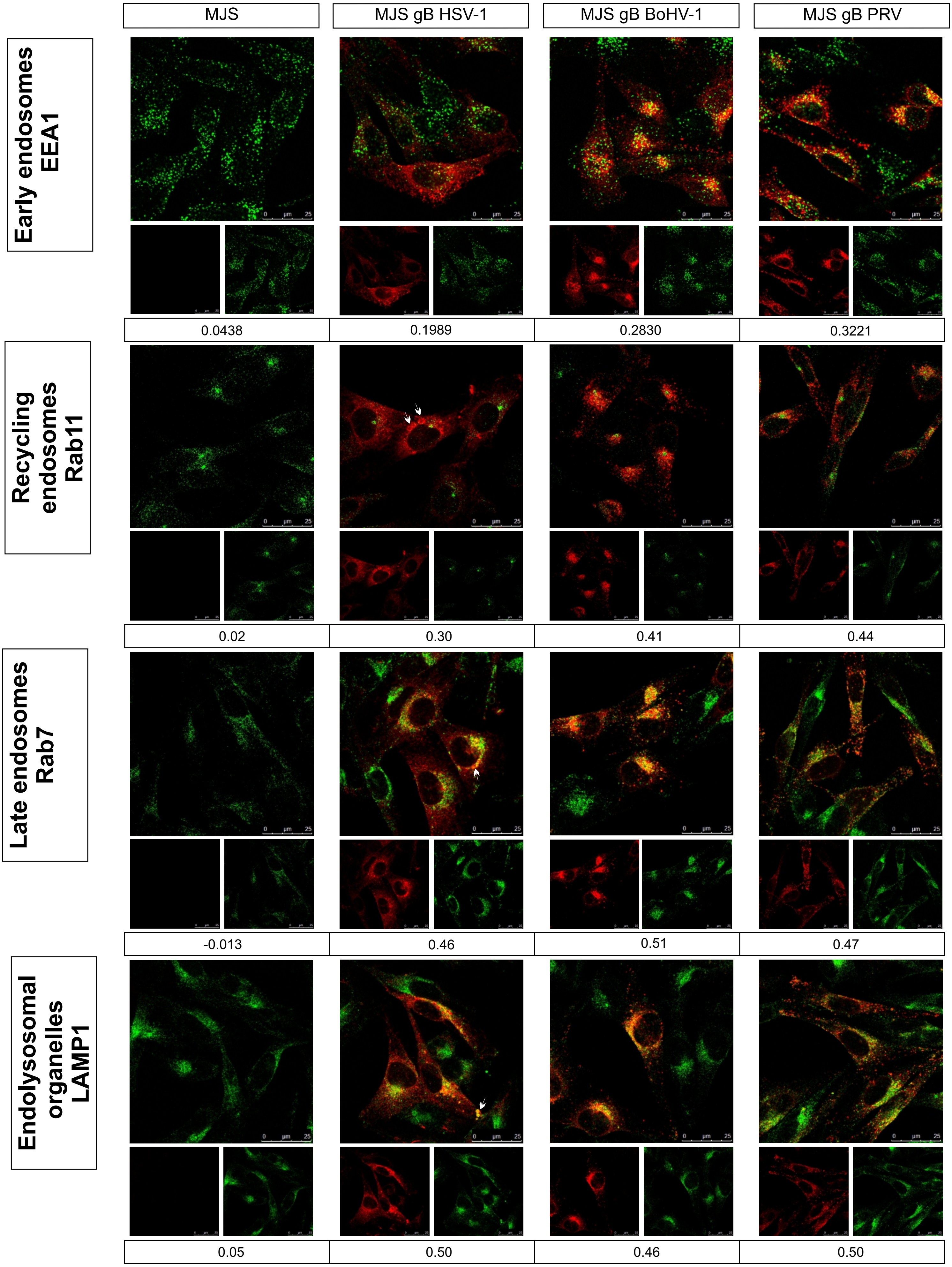

Supplementary Figure S3. Immunofluorescence-confocal laser-scanning microscopy analysis of endosomal/endolysosomal localization of gB homologs in the constructed MJS cell lines. HSV-1, BHV-1 or PRV gB was detected with specific mAb and Alexa 546-conjugated goat anti-mouse IgG; early endosome marker EEA1 or recycling endosome marker Rab11 or late endosome marker Rab7 or endolysosomal marker LAMP1 was detected in the same cells with rabbit mAb and Alexa 488-conjugated goat anti-rabbit IgG. MJSpuro cells were stained as controls. Co-localization was analyzed with the Pearson's coefficient, indicated below the pictures. Arrows indicate enlarged gB-positive vesicles.

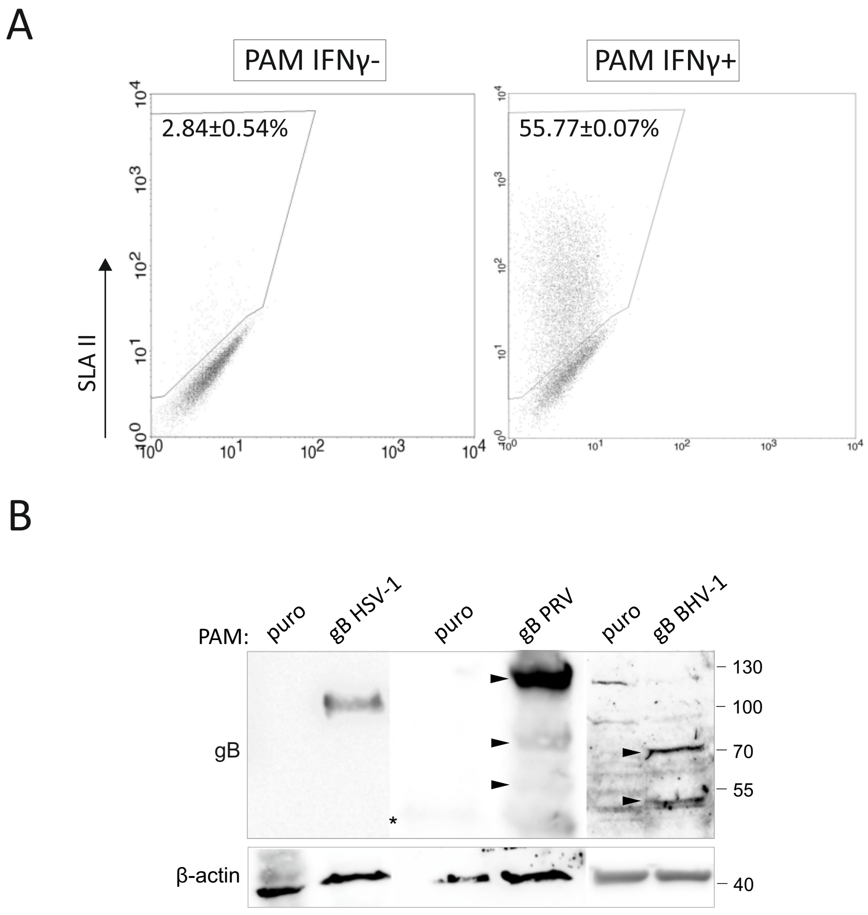

Supplementary Figure S4. (A) Flow cytometry analysis of endogenous swine MHC II (swine leukocyte antigen, SLA II) surface expression in porcine alveolar macrophages (PAM), clone 3D4/2. The cells were mock-treated or treated with 0,1 mg ml-1 of recombinant swine interferon (IFN)-γ for 36 hours prior analysis. The percentage of SLA II-positive cells from the triplicate analysis is indicated. (B) Immunoblotting detection of HSV-1, BoHV-1 or PRV gB in the lysates of constructed retrovirus-transduced and sorted PAM cell lines. Arrows indicate gBa, gBb, and gBc forms of PRV gB, and gBa, gBb fors of BoHV-1 gB. PAMpuro were constructed and analyzed as a control. β-actin was detected in cell lysates as a loading control. Size markers are in kilodaltons.

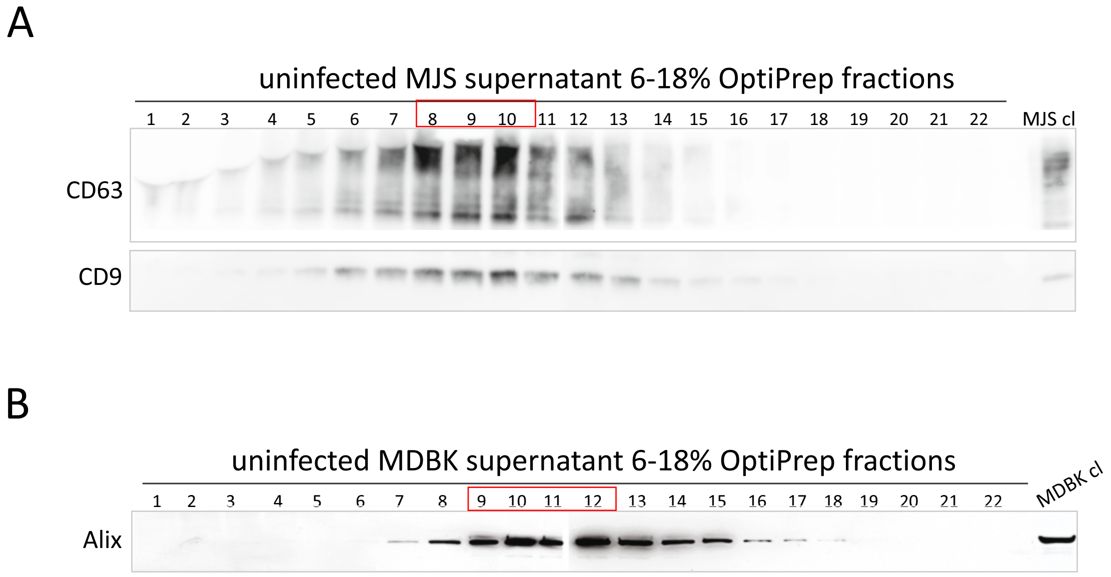

Supplementary Figure S5. OptiPrep gradient-purification of MJS (A) and MDBK EVs (B) derived from cell culture supernatants. 0.5 ml fractions were collected from 6-18% iodixanol (Optiprep) gradient by ultracentrifugation. 40 µl samples were analyzed in denaturing (for EVs marker Alix) or non-denaturing (for EVs markers CD63 and CD9) SDS-PAGE, and immunoblotted with specific mAbs. Cell lysates (cl) were analyzed as controls. The red frame indicates fractions with the peak EVs marker expression.

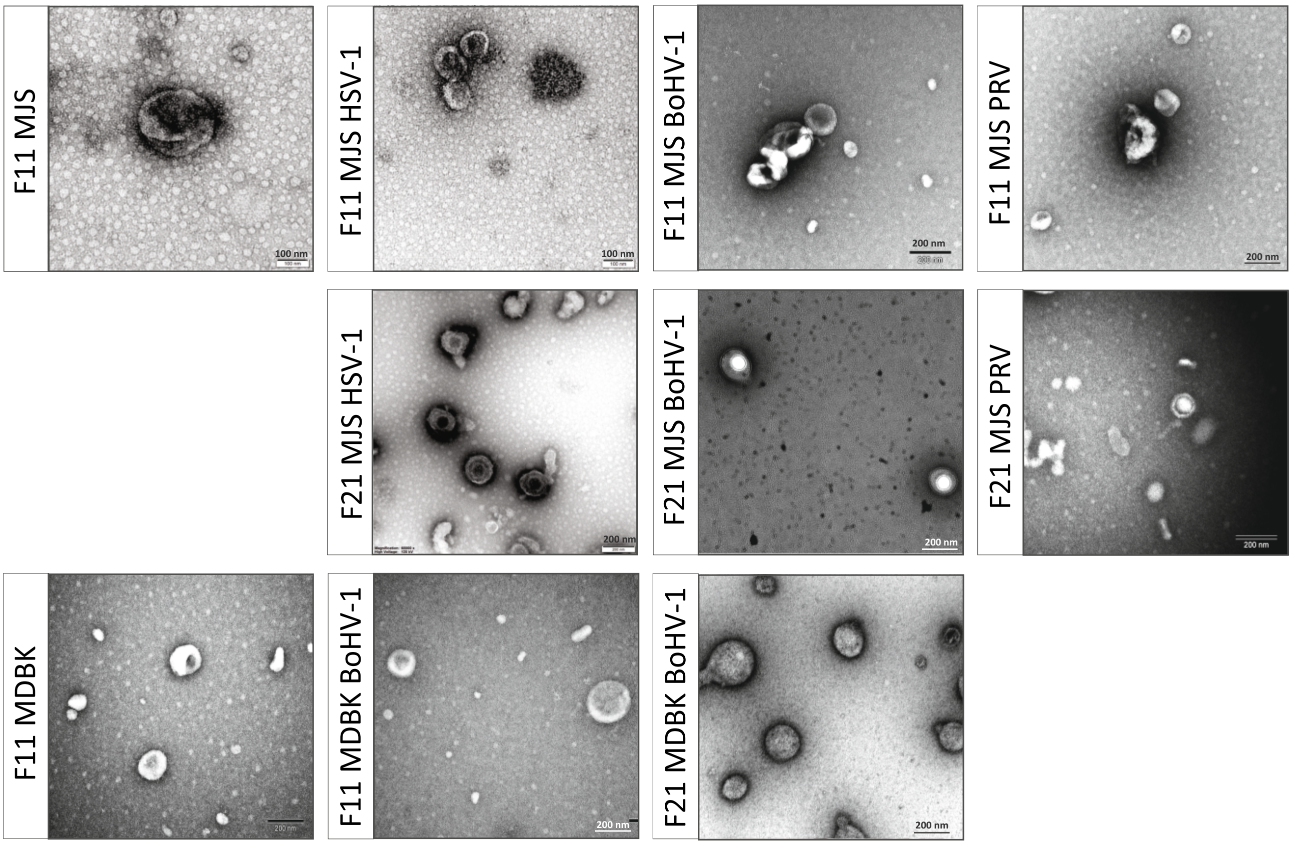

Supplementary Figure S6: Representative TEM images from the indicated OptiPrep gradient fractions from uninfected or virus-infected MJS or MDBK cell culture supernatants. Scale bar 100 nm or 200 nm, as indicated.
